# Retraction: A New Approach to Constructing Confidence Intervals for Population Means Based on Small Samples

**DOI:** 10.1371/journal.pone.0282629

**Published:** 2023-03-01

**Authors:** The PLOS ONE Editors

Following the publication of this article [1], concerns were raised regarding peer review integrity.

Upon review of the submission history for the manuscript, the editors found strong indications that the peer review process was compromised.

These concerns were not resolved following discussion with the authors.

In light of these concerns, the *PLOS ONE* Editors retract this article. We regret that the issues in this article were not identified prior to publication.

HCL and TL did not agree with the retraction and stand by the article’s findings. YX and CJH either did not respond directly or could not be reached.
